# Remediation of malachite green-laden wastewater by plant-loaded metal oxide nanocomposites

**DOI:** 10.1039/d5ra05373k

**Published:** 2025-09-11

**Authors:** Chisom Theresa Umeh, John Kanayochukwu Nduka, Kovo Godfrey Akpomie, Amalachukwu Ifeyinwa Obi, Nkeiru Magdalene Akanbi

**Affiliations:** a Environmental Chemistry and Toxicology Research Unit, Pure and Industrial Chemistry Department, Nnamdi Azikiwe University P.M.B 5025 Awka Anambra Nigeria umehchi871@gmail.com uc.umeh@unizik.edu.ng; b Department of Pure & Industrial Chemistry, University of Nigeria Nsukka Nigeria; c Department of Chemistry, University of the Free State Bloemfontein South Africa; d Department of Polymer Engineering, Federal University of Technology Owerri Nigeria

## Abstract

The aquatic environment is being endangered by contamination by organic dyes, which can lead to severe environmental pollution. This study presents the formation of nickel- and copper oxide-loaded *Bryophyllum pinnatum* (BP) nanocomposites (NiONPs-BP and CuONPs-BP) by a thermo-chemical precipitation process for sequestration of malachite green (MG) dye from a synthetically prepared aqueous solution. The synthesized nanomaterials were examined using FTIR, SEM, XRD, TGA and BET characterization techniques. The findings from the characterization techniques showed the distribution and impregnation of NiO and CuO on BP. The maximum adsorption capacities of NiONPs-BP and CuONPs-BP were 28.77 mg g^−1^ and 13.66 mg g^−1^, respectively. Isotherm and kinetic studies of catalytic MG dye removal are well described by the Freundlich and pseudo-second order models. The NiONPs-BP and CuONPs-BP nanocomposites exhibited higher regression values along with low error compared to BP, and proved better adsorbing materials. The spontaneous nature of MG adsorption was demonstrated by thermodynamic studies. For NiONPs-BP, Δ*S*° > 0 indicated increased disorder at the solid/liquid interface, while Δ*H*° > 0 confirmed the endothermic nature of adsorption. In contrast, adsorption on CuONPs-BP was also accompanied by increased disorder (Δ*S*° > 0) but proceeded through an exothermic process (Δ*H*° < 0). High removal efficiency was recorded after five successive reuse cycles, indicating remarkable stability and reusability of the nanocomposites. Therefore, NiONPs-BP and CuONPs-BP composites were suggested to be efficient adsorbing materials for remediating wastewater polluted with MG dye.

## Abstract 2

Nickel- and copper oxide-loaded *Bryophyllum pinnatum* (BP) nanocomposites (NiONPs-BP and CuONPs-BP) were prepared by a thermo-chemical precipitation process for sequestration of malachite green dye from a synthetically prepared aqueous solution.

## Graphical abstract

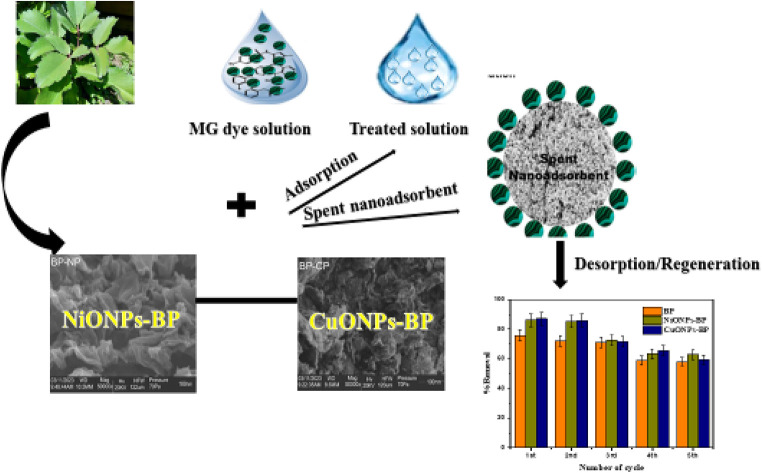

## 1. Introduction

In crystal form, malachite green dye (MG) is a shiny metallic material that dissolves readily in organic solvents and water. In aquaculture, the dye is frequently used as a disinfectant to prevent fungal and protozoal infections.^1^ Additionally, MG dye is used in the food industry as a coloring agent, in the textile industry to dye cotton, wool, silk, leather, paper, and acrylic, and in animal husbandry as a medical disinfectant.^2^ About 10–25% of the colors used in textile manufacturing are lost during the dyeing process, and 2–20% are discharged as effluents into the environment.^3,4^ Even in small quantities, the presence of effluents containing MG dye is undesirable, as it can cause serious water contamination. MG is considered a hazardous and cytotoxic dye as oral consumption through contaminated seafood has been linked to carcinogenic effects in humans.^5^ Additionally, it may cause dermatological problems including rashes and swelling, irritate the gastrointestinal and respiratory systems, and may harm human eyes.^6^ As a result, eating aquatic food contaminated with MG poses a serious risk to human health. The sequestration of MG using chemical, coagulation, biological and filtration processes is very challenging due to their high cost and lack of available adsorption mechanisms. Adsorption techniques prove to be more effective and versatile for the removal of MG dye from aqueous media.^7,8^ Some conventional adsorbents, including polymers, activated carbons, biomass, biochars, and zeolites, have been utilized widely in remediation studies of effluents, but there are documented drawbacks for most of them owing to either extended reaction time, low sorption capacity, cost of operation and production, disposal problems, *etc.*^9^ Hence, the development of novel adsorbents with improved dye removal efficiency, high stability and cost effectiveness is urgent. New developments in nanotechnology have made it possible to fabricate cost-effective, highly efficient and mechanically stable nano-adsorbents for wastewater treatment.^10^ Plant-loaded inorganic nickel and copper oxide nanomaterials show a good adsorption capacity and fast adsorption kinetics compared with conventional adsorbents.^11^ This results from their exceptionally high surface area and the presence of active functional groups on their surface, which facilitate interactions with water contaminants such as heavy metal ions and dye molecules.^12,13^ Recently, the adsorption of pollutants from wastewater onto nano-adsorbents like copper oxide nanoparticles (CuONPs) has attracted significant attention due to their high surface area, monoclinic structure, low cost, abundant active sites, narrow band gap, cytotoxicity, catalytic activity, favorable textural properties, antimicrobial properties, and efficient adsorption capacity.^14^ Nickel oxide has emerged as a significant functional material owing to its electrical, structural, optical, and catalytic capabilities.^15^ Because NiO is a p-type semiconductor with significant electrical and magnetic characteristics, it is being studied extensively.^16^

The resurrection plant, miracle leaf, divine plant, life plant, and marvel of the world are some of the common names for *Bryophyllum pinnatum* (Lam.) Oken (family: *Crassulaceae*).^17,18^ Tropical Africa, India, China, Australia, Madagascar, Asia, Hawaii, and tropical America all utilize this perennial herb extensively in traditional medicine.^19^ Its hemostatic and wound-healing qualities are well recognized.^20^ It has been used to treat allergies, burns, convulsions, diarrhoea, general debility, otitis, respiratory infections, skin ulcers, stomach ulcers, and a range of other illnesses and diseases in medicine.^19^ Alkaloids, triterpenes, glycosides, flavonoids, cardenolides, steroids, bufadienolides, and lipids are all abundant in *Bryophyllum pinnatum*, according to reports.^21^ A cheap and accessible adsorbent that comes from a biological source, miracle leaf is a strong contender for an effective and affordable substitute adsorbent for use in biosorption technology. Because of their different chemical components (such as volatile oils, steroids, and polyphenols and proteins) and their surface functional groups (such as carboxyl, hydroxyl, and carbonyl), the biomass materials indicated above can be regarded as potential natural adsorbents. An extensive review of the literature^22–31^ has shown that there is no documented research study on *Bryophyllum pinnatum* induced CuO and NiO nanoparticles in the context of wastewater management systems for malachite green pollutant. Prajapati and Mondal^32^ studied kinetic modelling and mass transfer for the removal of methylene blue using CuO loaded nanoporous activated carbon. Kedir *et al.*^33^ reported on the equilibrium performance of nanocellulose from sugarcane bagasse as a template for NiO nanoparticles for the uptake of organic dyes. The aim of this study is to evaluate the adsorption efficiency and to perform isotherm and kinetic modelling of prepared CuO- and NiO- nanoparticle-loaded *Bryophyllum pinnatum* for the removal of malachite green from aqueous solution. The novelty of the research is in the further explanation of the mechanism of adsorption and other underlying binding capacity for the biosorption of malachite green onto new biomass-synthesized nanocomposites, as well as the potential extent of reuse.

## 2. Materials and methods

### 2.1 Chemicals

Analytical grade NiSO_4_·6H_2_O, CuSO_4_·5H_2_O, sodium hydroxide (NaOH), hydrochloric acid (HCl), Cd(NO_3_)_2_·4H_2_O, malachite green dye (MG; C_23_H_25_N_2_Cl), and HNO_3_ were utilized without further purification.

### 2.2 Nanocomposite synthesis

The leaves of *Bryophyllum pinnatum* (BP) were harvested from the Orumba North region, located in the southeast of Nigeria. The vegetal material was dried for 2 d in sunlight and then was powdered using an electronic mixer. The green synthesis of nanocomposite adsorbents was conducted using a thermo-chemical precipitation process following the protocol described previously by Umeh *et al.*.^11^ Dissolution of 5 g of individual CuSO_4_·5H_2_O and NiSO_4_·6H_2_O in 100 mL of distilled water on a hot plate and stirring for half an hour at 30 °C to ensure the salts were completely dissolved were carried out. Then, a known amount of the produced BP was introduced to two separate beakers of salt solutions while being continuously stirred for 2 h. Some drops of 0.5 M NaOH were then added, and the mixture was stirred for a further 8 h, until the pH became alkaline. A color transition was observed following centrifugation and then oven drying was done at 70 degrees for two hours. The prepared adsorbents were designated as NiONPs-BP and CuONPs-BP composites.

### 2.3 Characterization

The characterization of CuO and NiONPs-*Bryophyllum pinnatum* was conducted using Fourier transform infrared (FTIR) spectroscopy (Bruker Tensor 27 IR), thermo-gravimetric analysis (TGA), a Mettler-Toledo TGA/SDTA851, scanning electron microscopy (SEM/EDS-JEOL-JSM-7600F), X-ray diffractometry (XRD-Rigaku D/MaxlllC, PW 1800), and surface area and porosity analysis (ASAP 2020 USA). A micrometric analyzer was used to obtain surface area and porosity measurements and the Brunauer–Emmett–Teller (BET) equation and the Barrett–Joyner–Halenda (BJH) method were utilized to estimate the relevant indicators.

### 2.4 Adsorption study

The adsorption of malachite green, BP, CuONPs-BP and NiONPs-BP was performed by a batch adsorption technique. In a typical procedure, a 1000 mg L^−1^ stock solution of the pollutant was prepared, followed by serial dilutions of the stock to concentrations of 10–50 mg L^−1^. Thereafter, 0.05 g of the respective adsorbent was added to 10 mL of a 50 mg L^−1^ pollutant solution at pH 7.0 and ultrasonicated for 60 min at 298 K, then filtered. The concentration of MG remaining in solution after adsorption was determined using UV measurements (wavelength of 320 nm). The effects of adsorbent dosage (0.05–0.25 g), sonication time (10–90 min), pH (3–11) and temperature (298–318 K) were studied by varying the factor of interest while keeping the others constant at the optimum conditions. The adsorption capacity and efficiency were estimated using eqn (1) and (2).1
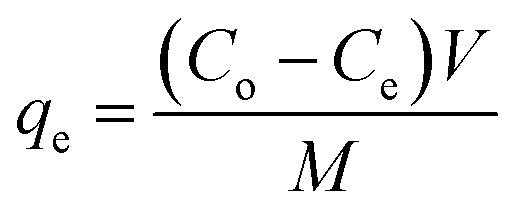
2
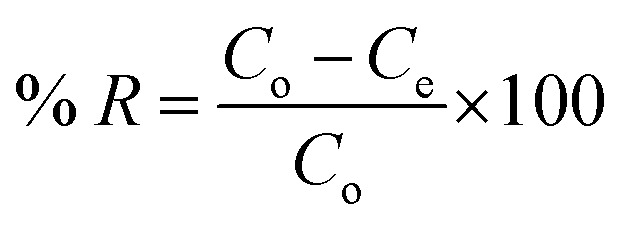


### 2.5 Regeneration experiment

The malachite green dye was desorbed from BP and the respective nanocomposites using 0.1 M HNO_3_ as an eluting agent. The desorption suspensions were stirred for 1 h at room temperature and then centrifuged at 4000 rpm for 30 min. The supernatants were subjected to UV for quantification of the desorbed MG dye. The separated adsorbents were washed with deionized water 4 times and used for resorption after oven drying at 105 °C for 3 h. The reusability potential of BP, NiONPs-BP and CuONPs-BP on the basis of calculated efficiency was ascertained for five periods of cycles by adsorption–desorption experiments. The desorption efficiency was computed using eqn (3).^34,35^3
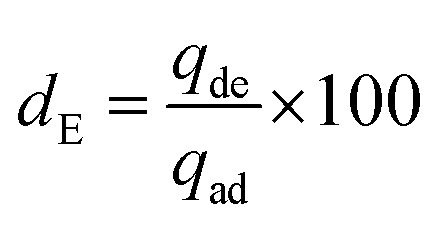
where *d*_E_ is the desorption efficiency (%), *q*_de_ is the desorbed concentration, and *q*_ad_ signifies the adsorbed concentration of the dye onto the prepared adsorbents.

## 3. Results and discussion

### 3.1 Characterization of BP, NiONPs-BP and CuONPs-BP

**Fig. 1:**
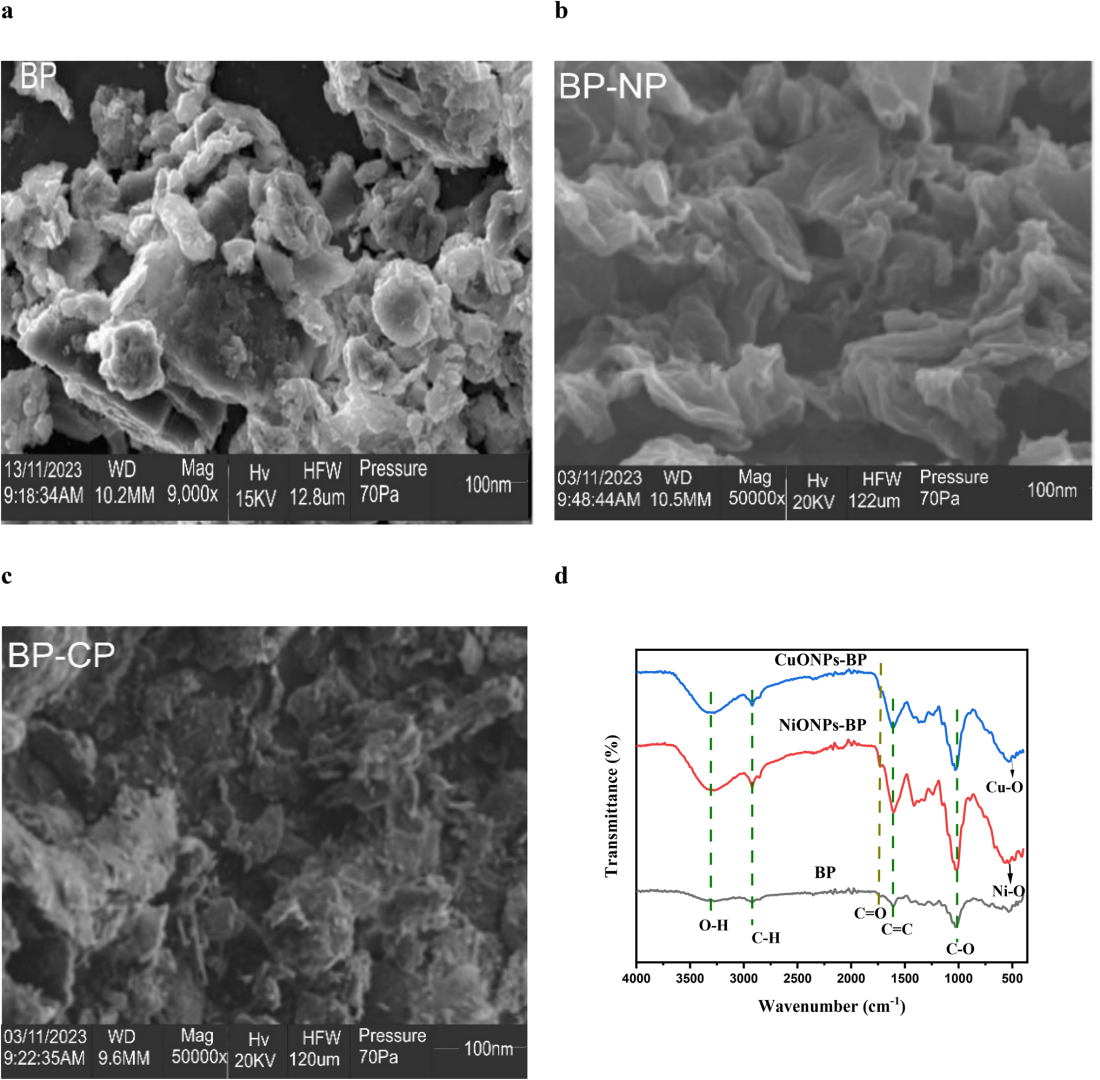
SEM of (a) BP, (b) NiONPs-BP and (c) CuONPs-BP at 100 nm, and (d) FTIR spectra of BP, NiONPs-BP and CuONPs-BP.

The surface morphology of BP, CuONPs-BP and NiONPs-BP was examined *via* SEM at specific magnification. Particles with a size of 100 nm showed a transformation from an irregular surface structure to one with more numerous porous surface sites, as shown in Fig. 1a–c. The surface structure of the BP was found to be rough and heterogeneous, with flaky microparticles, which suggested the potential presence of cavities. However, the adsorbent's microstructure was completely changed by impregnation with copper and nickel oxide. The channels permit the diffusion of the target pollutant molecules to the interior and exterior of the adsorbents.

The identification of functional groups in BP, CuONPs-BP and NiONPs-BP was achieved using FTIR spectroscopy as shown in Fig. 1d. As indicated for pristine BP, a weak characteristic peak was observed at around 3340 cm^−1^, which can be attributed to –O–H stretching vibration in phenol, alcohol, and adsorbed water.^36,37^ The peaks observed at around 2924, 1740, 1601, 1030 and 532 cm^−1^ could be assigned to the aliphatic C–H stretching vibration in alkane groups, C=O vibration in the carbonyl or carboxylic groups, C=C stretching of aromatic groups, C–O stretching vibration in carboxylic acids and esters and C–H bending in alkynes, respectively.^38,39^ After loading of CuO and NiO nanoparticles on BP, shifts in absorption bands from 3340 to 3341 and 3313 cm^−1^ (OH), 2924 to 2920 and 2926 cm^−1^ (C–H), 1740 to 1731 and 1732 cm^−1^ (C=O), 1601 to 1612 and 1609 cm^−1^ (C=C), and 1030 to 1026 and 1014 cm^−1^ (C–O) were observed. The bands at 571 and 574 cm^−1^ represent the stretching vibrations of inorganic CuO and NiO, respectively. Prajapati and Mondal^32^ found a similar situation for the adsorption of methylene blue using CuONPs, revealing a peak at 519 cm^−1^ that suggested Cu–O bonding. Kedir *et al.*^33^ reported the presence of a NiO (Ni–O) bond that could be the cause of the additional band at 580 cm^−1^. This showed that CuO and NiO impregnation actually occurred at the surface functional sites. Additionally, following CuO and NiO impregnation, an increase in the strength of these absorption bands was observed, suggesting that CuO and NiONPs had occupied these surface locations.

**Fig. 2:**
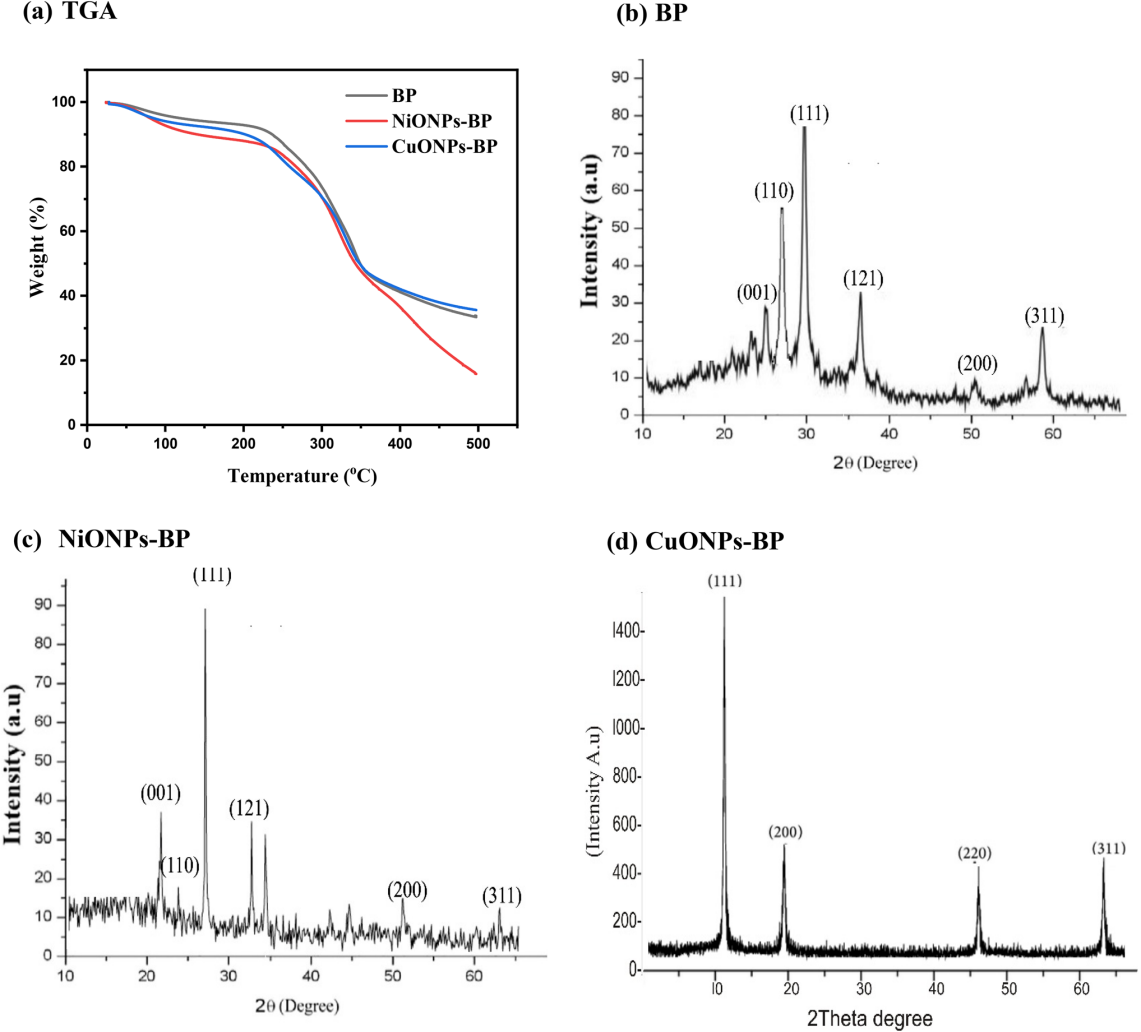
(a) TGA analysis and (b)–(d) XRD spectra of BP, NiONPs-BP and CuONPs-BP.

TGA analysis was used to determine the thermal behavior of BP and its metal oxide nanocomposites, as seen in Fig. 2a. From 100 °C to 400 °C, the investigated raw and nanocoated adsorbents showed a considerable weight loss, indicating the removal of moisture and the breakdown of organic matter.^40^ The low weight loss observed for CuONPs-BP and NiONPs-BP at the first and second stages of the curve with temperature indicates that the impregnation with copper oxide and nickel oxide nanoparticles increases the thermal stability of BP; whereas at stage 3, a noticeable weight loss was observed for NiONPs-BP, which could be attributed to a combination of nanoparticle decomposition and changes to the original sample composition. In addition, it is interesting to note that the degradation of BP and its respective nanocomposites follows a similar mechanism.

The XRD spectra of BP, CuONPs-BP and NiONPs-BP are shown in Fig. 2b–d. The mixture of broad and sharps peaks at 2*θ* = 25.39°, 28.85°, 30.97°, 35.62°, 50.56° and 59.02°, which correspond to the (001), (110), (111), (121), (200) and (311) planes, is consistent with BP. For the composite NiONPs-BP, the appearance of the characteristic metal oxide diffractions at 2*θ* values of 21.38°, 24.67°, 27.89°, 33.13°, 52.7° and 63.68° was observed, corresponding to the six structural planes of (001), (110), (111), (121), (200) and (311), respectively. In the case of the CuONPs-BP adsorbent, diffractions at 2*θ* values of 12.41°, 19.33°, 46.55° and 64.08° corresponding to structural planes of (111), (200), (220) and (311) were estimated. The effective impregnation of CuO and NiO onto BP in the hybrid adsorbent is confirmed by the presence of sharp diffraction peaks in the nanocomposite compared to broad peaks in the native plant. Prajapati and Mondal^32^ reported a similar XRD pattern for the synthesized CuONPs. The sizes of the NiONPs-BP and CuONPs-BP nanocomposites were 57.01 and 62.35 nm, compared to the 3.30 nm crystalline size of BP, which also confirmed successful fabrication. The peaks for nickel oxide and copper oxide nanoparticles occur at 28.34° and 32.49°.

**Fig. 3:**
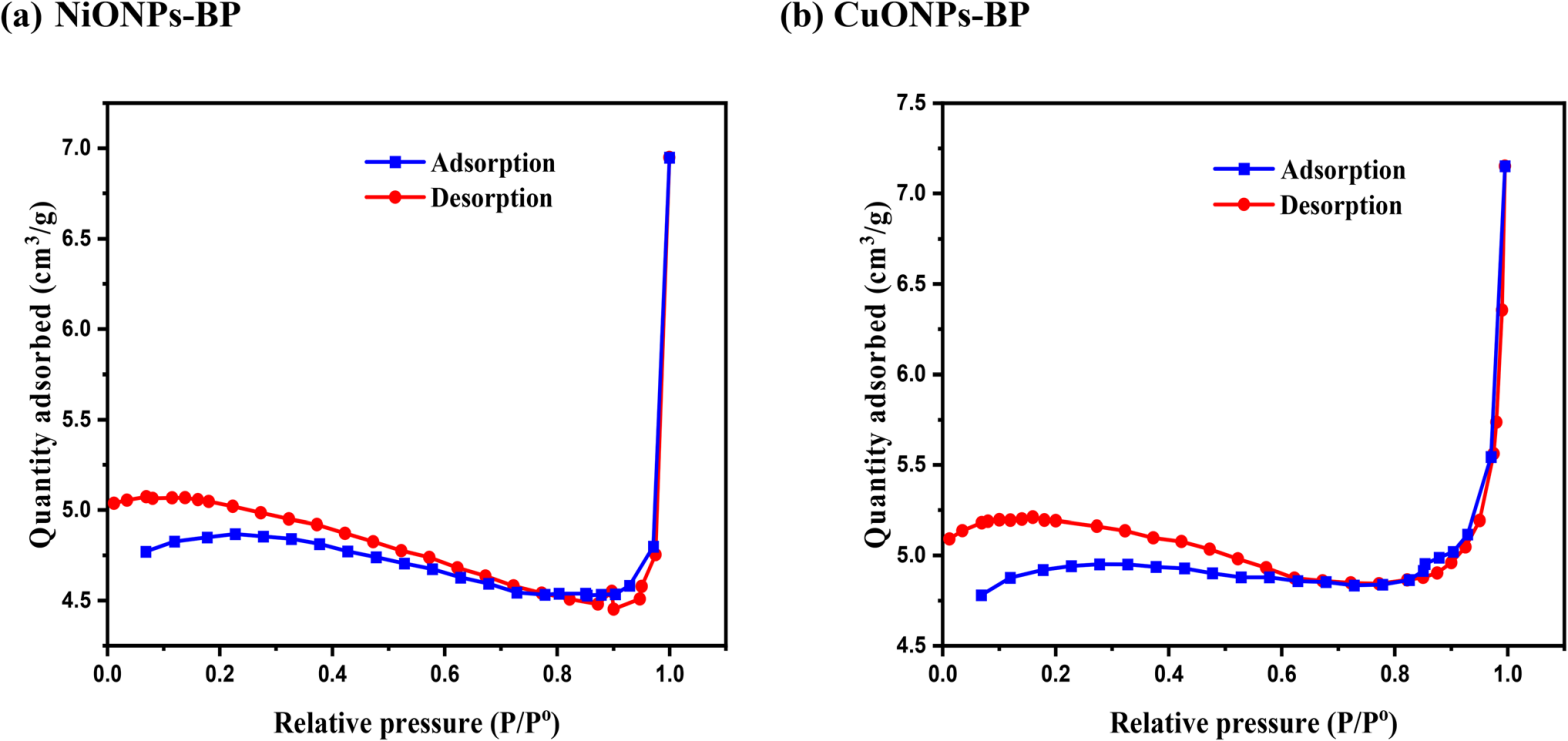
Nitrogen adsorption–desorption isotherms for (a) NiONPs-BP and (b) CuONPs-BP.

The N_2_ adsorption–desorption isotherms and pore volume distributions of BP and the NiONPs-BP and CuONPs-BP nanocomposites are shown in Fig. 3. The BET surface areas of the BP, NiONPs-BP, and CuONPs-BP adsorbents were found to be 0.44, 0.41 and 0.91 m^2^ g^−1^, respectively. A total pore volume of 0.00042 cm^2^ g^−1^ and pore size of 6.25 nm were obtained for CuO loaded composites according to the Barrett–Joyner–Halenda method (BJH), which are higher than 0.00036 cm^2^ g^−1^ and 5.60 nm for the NiO impregnated composite and 0.00038 cm^2^ g^−1^ and 6.19 nm for BP. Compared to the NiONPs-BP composite, the improved pore characteristics would probably favor the adsorption of target pollutant molecules onto CuONPs-BP. It is crucial to remember that a decreased surface area does not necessarily translate to a lower adsorption capability of the adsorbent.^41^ Furthermore, a mesoporous structure is demonstrated by the presence of an H3-type hysteresis loop for CuONPs-BP and NiONPs-BP, which correlates to a type IV isotherm.^42^ For effective adsorption, metal ions, dye, and medicinal molecules could easily diffuse through the pores of the adsorbents due to their mesoporosity.^43^

### 3.2 Point of zero charge (pHpzc) and effect of pH

**Fig. 4:**
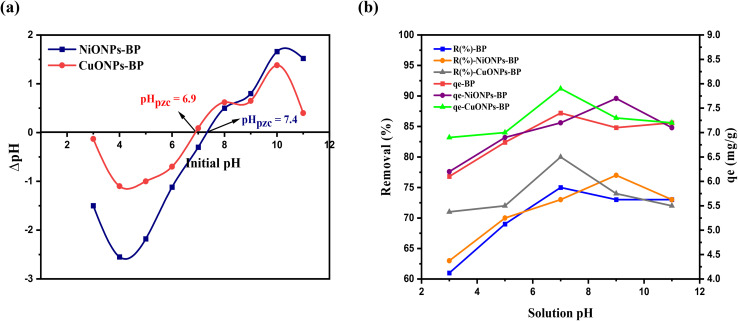
(a) The pH point of zero charge and (b) effect of pH on adsorption of MG onto BP, NiONPs-BP and CuONPs-BP.

The pH at which the total number of charges at the adsorbent surface is zero is the pHpzc. It is a vital parameter when the adsorption mechanism involves electrostatic interaction. It is noted that when pH < pHpzc, the adsorbent surface is positively charged, and it is negatively charged when pH > pHpzc. The pHpzc values of NiONPs-BP and CuONPs-BP were identified to be 7.4 and 6.9, respectively, as shown in Fig. 4a, and as such, the nanocomposite surfaces are positive for pH < 6.9 and 7.4, but negative for pH > 6.9 and 7.4. MG adsorption is favorable when the surface is negatively charged; for this reason, the pH must be greater than the evaluated pHpzc.^15^

The effect of pH on MG adsorption was investigated using an adsorbent mass of 0.1 g, and an initial dye concentration of 50 mg L^−1^ at 25 °C for an equilibrium time of 60 min. According to Lin *et al.*^44^ the pH affects the surface charges, level of ionization, and dissociation of functional groups at the adsorbent sites. As shown in Fig. 4b, the amount of adsorption of MG onto BP, NiONPs-BP, and CuONPs-BP was very low when the pH of the solution was extremely acidic (pH 3 to 5). Adsorption increased with increasing pH, reaching a maximum at pH 7, and decreased thereafter. About 61%, 63% and 70% of MG at pH 3 were adsorbed by BP, NiONPs-BP and CuONPs-BP, which increased up to 75%, 77% and 80% at pH 7, and reduced with further pH increase. The lower sequestration of MG at acidic pH is due to the abundance of hydrogen ions that tend to compete with cationic MG on empty adsorbent surface sites, leading to electrostatic repulsion. Meanwhile, on an alkaline medium, the adsorbent surface is negative, permitting and improving the adsorption of MG dye due to electrostatic attractions.

### 3.3 Effect of dosage and contact time

When the doses of BP, NiONPs-BP and CuONPs-BP were increased in the aqueous solution of the target pollutant, the removal percentage increased, and maximum removal was achieved at a dose of 0.1 g. The reason behind the increased removal efficiency is that when the dose of the adsorbents increases, both the number of active sites and the surface area of the adsorbents increase, which are essential factors for high adsorption.^45^ When the individual doses of BP and the NiONPs-BP and CuONPs-BP nanocomposites were in excess of 0.1 g, the removal % of MG remained unchanged due to the unavailability of adsorbate molecules in the aqueous solution. This phenomenon could be attributed to a higher rate of collision among the adsorbent particles, which led to their aggregation or the overlapping of active adsorption sites, thereby resulting in a shortage of unoccupied adsorption sites.^46^ However, the impregnation of the reported metal oxide on BP improved the removal efficiency of the MG dye. Abate *et al.*^47^ experienced a similar decrease in MG removal when the mass of activated carbon of *khat* exceeded 0.5 g. Again, the removal percentage of dye reduced after treatment with 0.05 g of TiO_2_-PAM-CS as reported by Ehsan *et al.*^48^

**Fig. 5:**
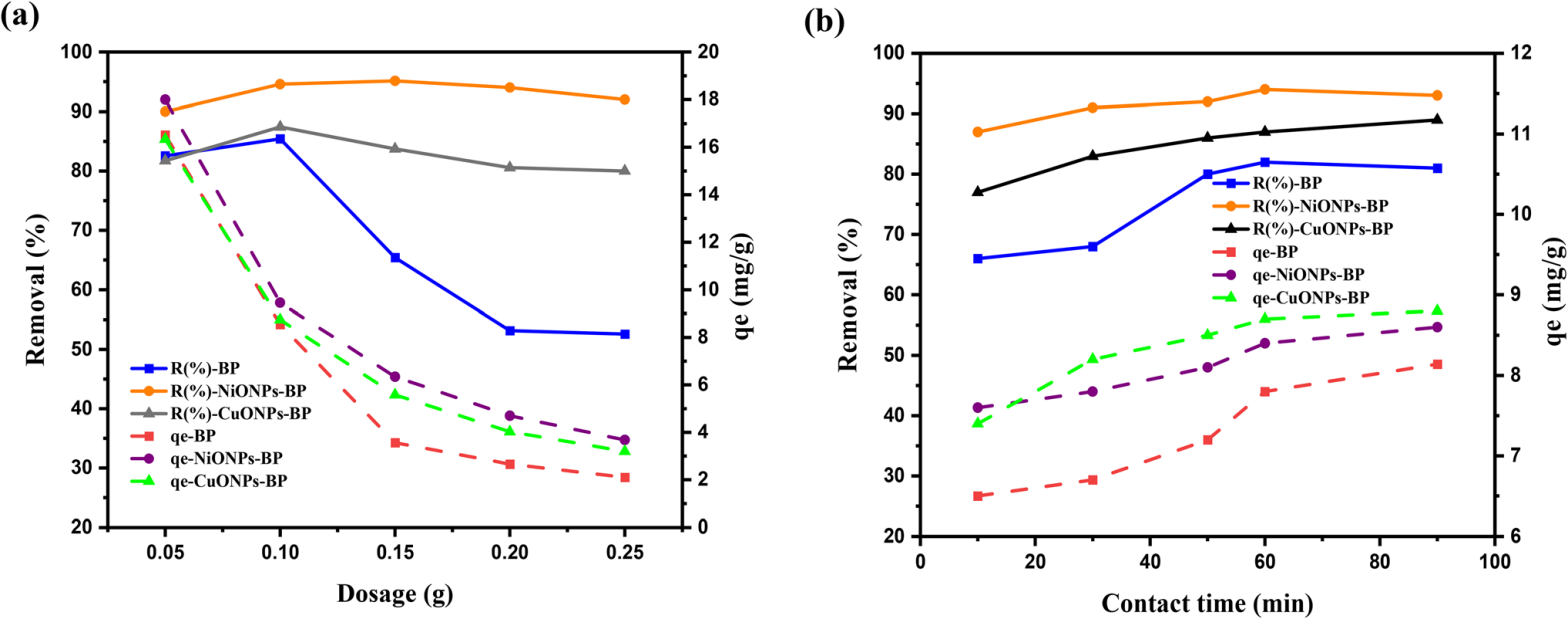
Effect of (a) dosage and (b) contact time on the adsorption of MG onto BP, NiONPs-BP and CuONPs-BP.

The time it takes for the solid adsorbents to adsorb the adsorbates from the liquid until equilibrium is attained is known as the equilibrium time for the adsorption of MG dye. The percentage removal gradually increased until it reached equilibrium after 60 min for all the studied adsorbents. The amount of adsorbed MG dye rapidly increased at first and as the process continues, the sites are quickly occupied by adsorbate ions/molecules until equilibrium is achieved. This is probably because of the initial availability of a large number of unoccupied active sites, which become saturated as time progresses.^49^ A similar trend was observed for the adsorption capacity as can be seen in Fig. 5b. The loading of CuO and NiO nanoparticles onto BP increased the removal efficiency of the dye.

### 3.4 Adsorption isotherms

**Fig. 6:**
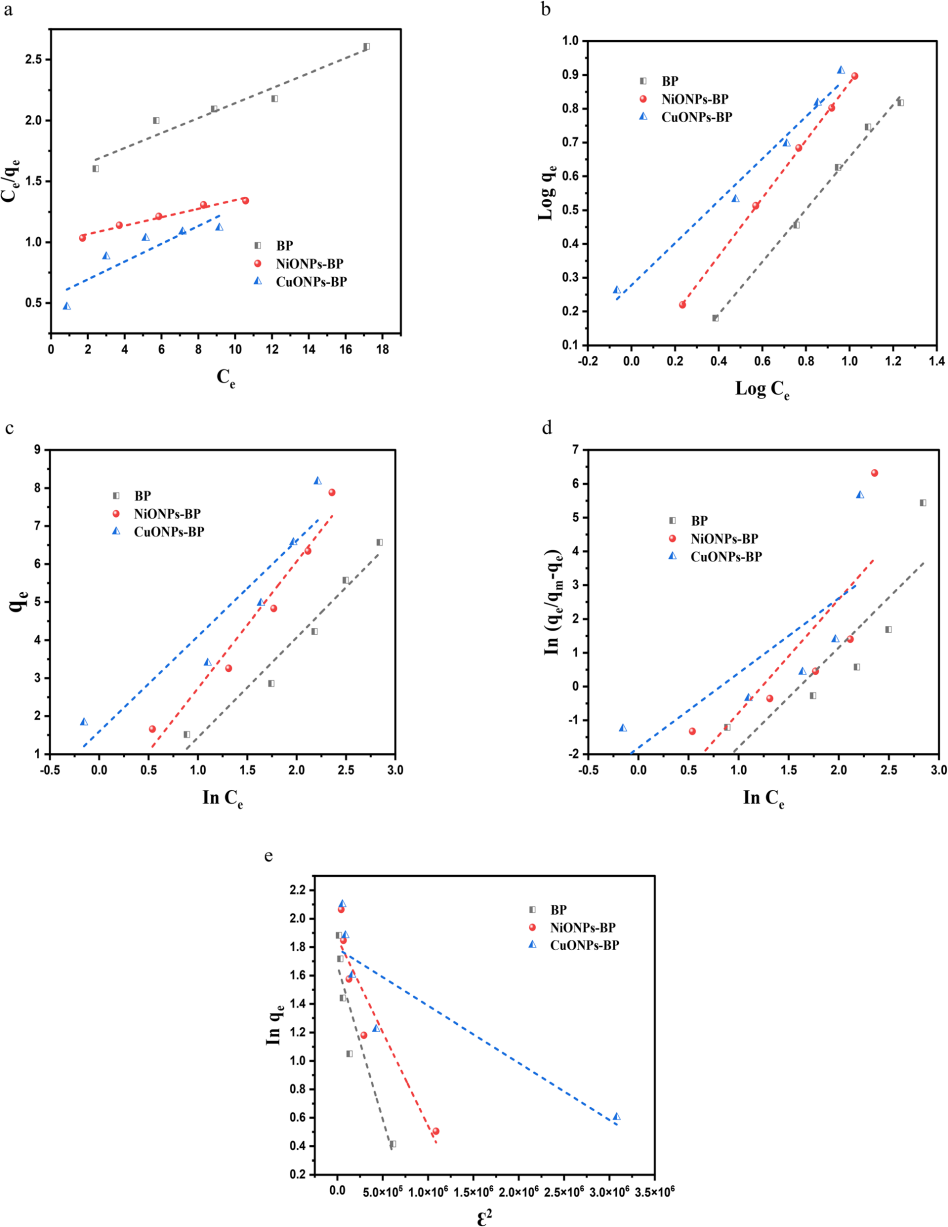
(a) Langmuir, (b) Freundlich, (c) Temkin, (d) Sips and (e) Dubinin–Radushkevich plots for MG uptake onto BP, NiONPs-BP and CuONPs-BP.

The adsorption isotherms show the interaction of MG dye with adsorbents and give information on the adsorption capacities of the studied adsorbent materials.^50^ The use of models to fit the experimental data was evaluated using regression coefficients (*R*^2^ values) and error analysis. The model and error analysis equations are given in the supplementary file. The adsorption data of MG on BP, NiONPs-BP and CuONPs-BP were found to fit well to the Langmuir, Freundlich, and Temkin models,^51^ with *R*^2^ values for MG of 0.9421, 0.9952, and 0.9608 for BP; 0.9698, 0.9997 and 0.9546 for NiONPs-BP; and 0.8061, 0.9828 and 0.8856 for CuONPs-BP; and low calculated error as shown in Fig. 6 and Table 1. However, the Freundlich model could better describe the adsorption behavior of MG on BP and its respective nanocomposites, which confirms the uptake of MG dye by the heterogeneous surface.^52^ It can also be inferred that *n* values greater than 1 and *R*_L_ values between 0 and 1 depicted favorable adsorption of MG onto adsorbent materials. The loading of the nickel oxide composites on BP improved the dye uptake more than copper oxide, which results in a higher *R*^2^ value for the Freundlich model. The fit of the Freundlich model favors the multilayered MG dye binding on the heterogeneous surface of the prepared NiONPs-BP and CuONPs-BP nanocomposites. Furthermore, the obtained *E* (kJ mol^−1^) values of the D–R isotherm were less than 8 kJ mol^−1^, which confirms the physisorption of MG.

**Table 1:** Isotherm parameters for the uptake of MG on BP, NiONPs-BP and CuONPs-BP

Model	Parameters	BP	NiONPs-BP	CuONPs-BP
Langmuir	*K*_L_ (L mg^−1^)	0.094	0.035	0.040
	*q*_m_ (mg g^−1^)	16.26	28.77	13.66
	*R* ^2^	0.9421	0.9698	0.8061
	*R*_L_	0.302	0.522	0.488
	SSE	0.099	0.035	1.236
	EABS	0.595	0.367	2.284
	*χ* ^2^	0.026	0.006	0.300
Freundlich	*K*_F_ (L g^−1^)	0.762	1.053	1.896
	*n*	1.291	1.170	1.604
	*R* ^2^	0.9952	0.9997	0.9828
	SSE	0.207	0.010	0.646
	EABS	0.799	0.184	1.509
	*χ* ^2^	0.036	0.002	0.113
Temkin	*K*_T_ (L g^−1^)	0.635	0.835	1.886
	*B* (kJ mol^−1^)	2.63	3.33	2.51
	*R* ^2^	0.9608	0.9546	0.8856
	SSE	0.648	1.096	2.877
	EABS	1.708	2.172	3.371
	*χ* ^2^	0.236	0.306	0.714
Sips	1/*n*	2.95	3.36	2.21
	*q*_m_ (mg g^−1^)	6.60	7.90	8.20
	*K*_S_ (L mg^−1^)	0.008	0.016	0.163
	*R* ^2^	0.7505	0.6607	0.5991
	SSE	4.014	7.607	10.131
	EABS	3.969	5.460	6.335
	*χ* ^2^	1.343	2.018	2.652
D–R	*K*_DR_ (mol^2^ kJ^−2^)	2.16 × 10^−6^	1.32 × 10^−6^	4.02 × 10^−7^
	*q*_m_ (mg g^−1^)	5.32	6.43	5.98
	*E* (kJ mol^−1^)	0.481	0.615	0.112
	*R* ^2^	0.8553	0.8817	0.7848
	SSE	3.924	5.073	9.121
	EABS	3.729	4.099	5.483
	*χ* ^2^	0.879	0.910	1.634

A comparison of the current findings with various adsorbents for monolayer adsorption capacity for the removal of MG dye is presented in Table 2. Although the prepared adsorbents in the present study gave lower adsorption capacity than most adsorbents in the literature, their uptakes were higher than *Catha edulis* activated carbon (5.62 mg g^−1^)^47^ and PMMA/GO-Fe_3_O_4_ (11.01 mg g^−1^).^53^ On the other hand, the uptake capacity of the NiONPs-BP composite was higher than rice husk (18.10 mg g^−1^),^54^ Fe–Zn bimetallic nanoparticles (21.74 mg g^−1^),^55^ and corn stalk (16.47 mg g^−1^).^56^ The core benefit in the application of the prepared adsorbents is their abundance and eco-friendly nature for the remediation of wastewater contaminated with MG dye.

**Table 2:** Comparison of various adsorbents for adsorptive removal of malachite green

Adsorbents	Pollutants	*q*_e_ (mg g^−1^)	References
GO-CEL-Cu nanocomposite	MG	202.8	1
Water chestnut	MG	136.46	34
Corn silk	MG	18.38	35
*Catha edulis* activated carbon	MG	5.62	47
rGO/γ-Fe_2_O_3_	MG	64.26	52
PMMA/GO-Fe_3_O_4_	MG	11.01	53
Rice husk	MG	18.10	54
Fe–Zn bimetallic nanoparticles	MG	21.74	55
Corn stalk	MG	16.47	56
Neem leaf	MG	66.72	57
*Azolla pinnata*	MG	292.1	58
*Luffa aegyptiaca* peel	MG	161.29	59
Corn cob	MG	64.52	60
*Bryophillum pinnatum* (BP)	MG	16.26	This study
NiONPs-BP	MG	28.77	This study
CuONPs-BP	MG	13.66	This study

### 3.5 Kinetic study

**Fig. 7:**
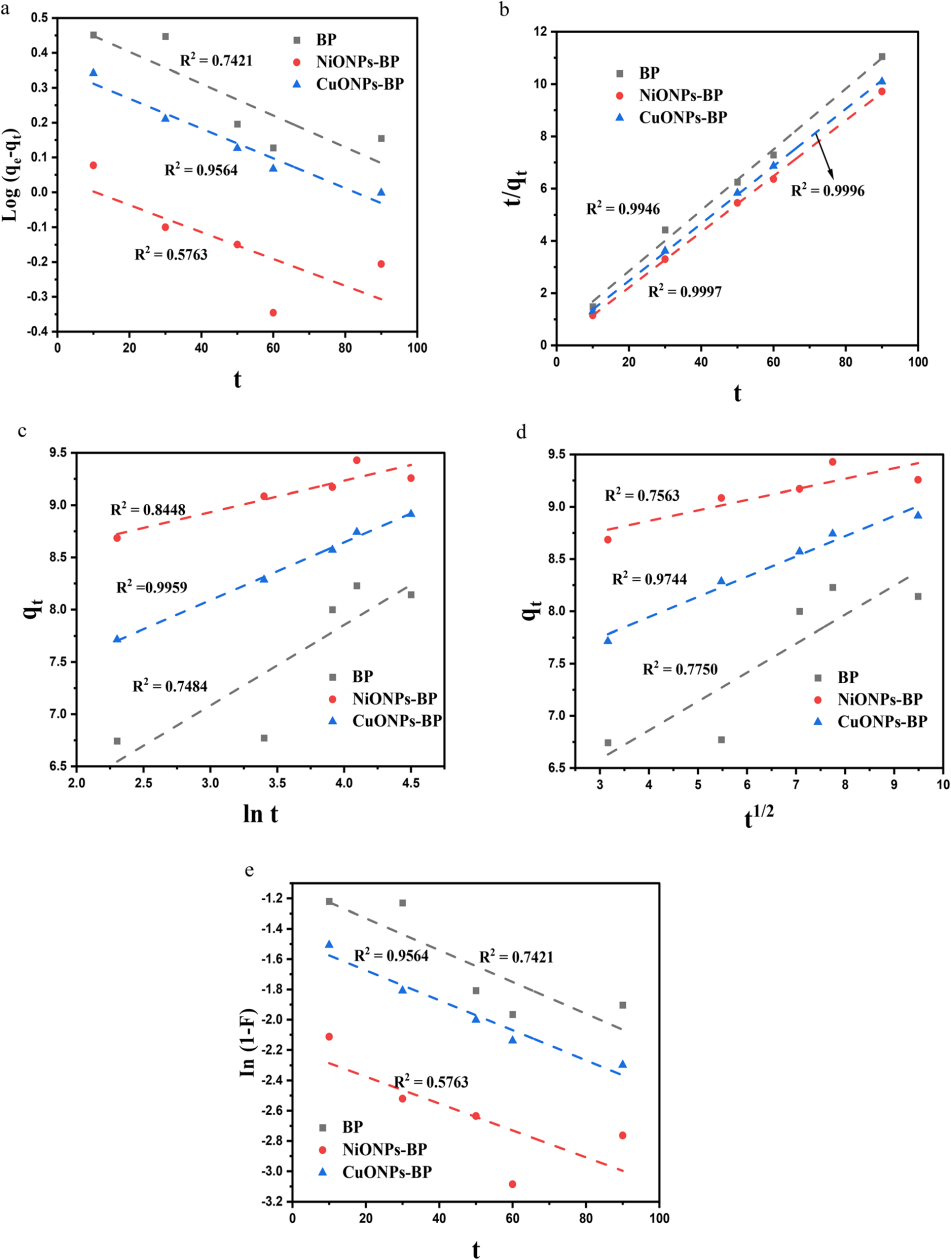
(a) PFO, (b) PSO, (c) Elovich, (d) IPD and (e) LFD plots for MG uptake onto BP, NiONPs-BP and CuONPs-BP.

To estimate the rate at which MG binds to BP, NiONPs-BP, and CuONPs-BP and to clarify the mechanism of the adsorption process, the rate of adsorption is calculated. Several kinetic models, such as pseudo-first-order (PFO), pseudo-second-order (PSO), and Elovich, were employed at equilibrium concentration to evaluate adsorption control, whereas liquid film diffusion (LFD) and IPD models were also used to model the experimental data.^61^ The kinetic equations are shown in the supplementary file.^62^ The kinetics plots and findings are displayed in Fig. 7 and Table 3. As can be seen, for the studied MG pollutant, the BP and respective nanocomposites equilibrium data follow the PSO model. The *R*^2^ and residual sum square (RSS) values of BP, NiONPs-BP and CuONPs-BP were 0.9946 and 0.272, 0.9997 and 0.017, and 0.9996 and 0.017. The numbers are close to one another, particularly for the produced nanocomposites, and the *q*_e_ derived from the experiments matches the computational *q*_e_, suggesting that the findings are more in line with this model. This also confirmed a better adsorption process utilizing metal oxide nanocomposites. The agreement of the experimental data with the PSO models suggests that MG dye adsorption occurred by chemisorption interaction of the adsorbate on the adsorbent active sites. Thus, the dye molecules bind to the active sites of the adsorbent by covalent bonding, which corresponds to the chemisorption-controlled process (John *et al.*, 2024).^46^ The adsorption of MG dye followed the Freundlich isotherm and pseudo-second order models, which is consistent with literature reports by Dada *et al.*^62^ and Debnath and Das.^63^ The diffusion mechanism of the adsorption procedure was confirmed by using the intraparticle diffusion and liquid film diffusion models. To investigate the steps involved in MG dye adsorption, the intraparticle diffusion model (IPD) was studied. It is clear from the linear parts in the plots that the uptake of MG was a two-stage process. The first stage involves the movement of MG molecules from the bulk film to the exterior surface of the BP and nanocomposites as a result of hydrogen bonding, whereas in the second stage of diffusion, the dye molecules diffuse from the exterior heterogeneous surface to the interior pores of the adsorbent due to van der Waals forces.^53^ Therefore, the kinetics of dye adsorption are controlled by the second stage process, as shown in Fig. 7d. The higher regression values of the IPD model when compared to the LFD kinetic model indicated that the IPD model describes the major diffusion mechanism for MG removal onto BP, NiONPs-BP and CuONPs-BP. This is because the contact between the MG and the studied adsorbents occurred in an agitated continuous adsorption system that promotes efficient dye molecule diffusion from the surface to the internal pores of the adsorbent sites.^41^

**Table 3:** Kinetic parameters for the adsorption of MG on BP, NiONPs-BP and CuONPs-BP

Model	Parameters	BP	NiONPs-BP	CuONPs-BP
Pseudo-first order	*q*_eexp_ (mg g^−1^)	9.57	9.88	9.91
	*k*_1_ (min^−1^)	0.011	0.009	0.010
	*q*_ecal_ (mg g^−1^)	3.12	1.10	2.26
	*R* ^2^	0.7421	0.5763	0.9564
	RSS	0.027	0.040	0.003
Pseudo-second order	*k*_2_ (mg g^−1^ min^−1^)	0.026	0.143	0.043
	*qe*_cal_ (mg g^−1^)	8.60	9.38	9.12
	*R* ^2^	0.9946	0.9997	0.9996
	RSS	0.272	0.017	0.017
Elovich	*α* (g min^2^ mg^−1^)	379.96	1.22 × 10^11^	6.00 × 10^4^
	*β* (g min mg^−1^)	1.30	3.33	1.80
	*R* ^2^	0.7484	0.8448	0.9959
	RSS	0.571	0.048	0.004
Intraparticle diffusion	*K*_id_ (mg g min^−1/2^)	0.28	0.10	0.19
	*C*	5.75	8.46	7.17
	*R* ^2^	0.7750	0.7563	0.9744
	RSS	0.510	0.075	0.023
Liquid film diffusion	*K*_fd_	0.011	0.009	0.010
	*D*	−1.121	−2.197	−1.477
	*R* ^2^	0.7421	0.5763	0.9564
	RSS	0.141	0.214	0.016

### 3.6 Thermodynamic study

**Fig. 8:**
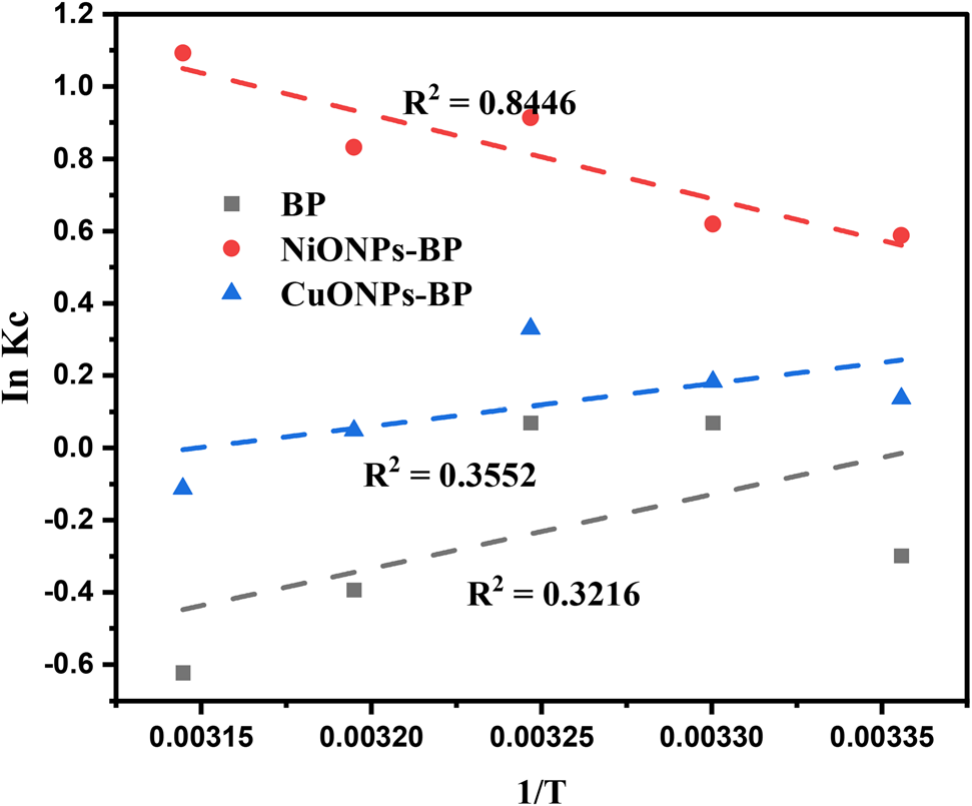
Van ’t Hoff plot for the adsorptive removal of MG by BP, NiONPs-BP and CuONPs-BP.

In order to comprehend how the adsorption mechanism affects temperature changes, the adsorption of MG molecules by BP, and the NiONPs-BP and CuONPs-BP nanocomposites, was investigated at temperatures between 298 and 318 K, as indicated in Fig. 8 and Table 4. The equation used is given in the supplementary file. The negative Δ*G*° showed that MG spontaneously adsorbs on both raw and nano-adsorbents at all temperatures. The observed decrease in Δ*G*° values with rising temperature indicates that MG dye adsorption onto NiONPs-BP and CuONPs-BP nanocomposites is more favourable at higher temperatures. An increase in disorder at the dye/NiONPs-BP solid–solution interface during the process was indicated by the positive Δ*S*° value of 69.22 J mol^−1^ K^−1^. In contrast, the negative Δ*S*° values of entropy obtained for BP and CuONPs-BP indicate that the adsorption process was accompanied by decreased randomness. Dahmani *et al.*^15^ reported negative Δ*G*° and positive Δ*S*° values for MG uptake onto the NiO adsorbent. The positive Δ*H*° values indicated that the adsorption of MG onto NiONPs-BP was endothermic, even though the adsorption slightly decreased as the temperature increased. NiONPs-BP was shown to be a better adsorbing nanomaterial than CuONPs-BP following its higher regression value of 0.8446 compared to 0.3552. A similar endothermic adsorption of paracetamol and thiazolyl blue dye onto CuONPs has been reported.^14^ In contrast, the adsorption of MG dye onto BP and CuONPs-BP displayed negative Δ*H*° values that depict an exothermic process. The obtained Δ*H*° values were less than 40 kJ mol^−1^, which conforms to physical adsorption^64^ that involved electrostatic and van der Waals attractions. This physical adsorption would be favorable for easy desorption of the MG dye from the biomass loaded nanoparticle.

**Table 4:** Thermodynamic study of MG adsorption onto BP, NiONPs-BP and CuONPs-BP

Adsorbent	Temperature	Δ*G*° (kJ mol^−1^)	Δ*H*° (kJ mol^−1^)	Δ*S*° (J mol^−1^ K^−1^)	*R* ^2^
**Malachite green dye**					
BP	298 K	0.739	−17.074	−57.41	0.3216
	303 K	−0.177			
	308 K	−0.180			
	313 K	1.023			
	318 K	1.645			
NiONPs-BP	298 K	−1.456	19.238	69.22	0.8446
	303 K	−1.562			
	308 K	−2.338			
	313 K	−2.167			
	318 K	−2.889			
CuONPs-BP	298 K	−0.338	−9.725	−30.62	0.3552
	303 K	−0.459			
	308 K	−0.845			
	313 K	−0.127			
	318 K	0.297			

### 3.7 Desorption and reuse

**Fig. 9:**
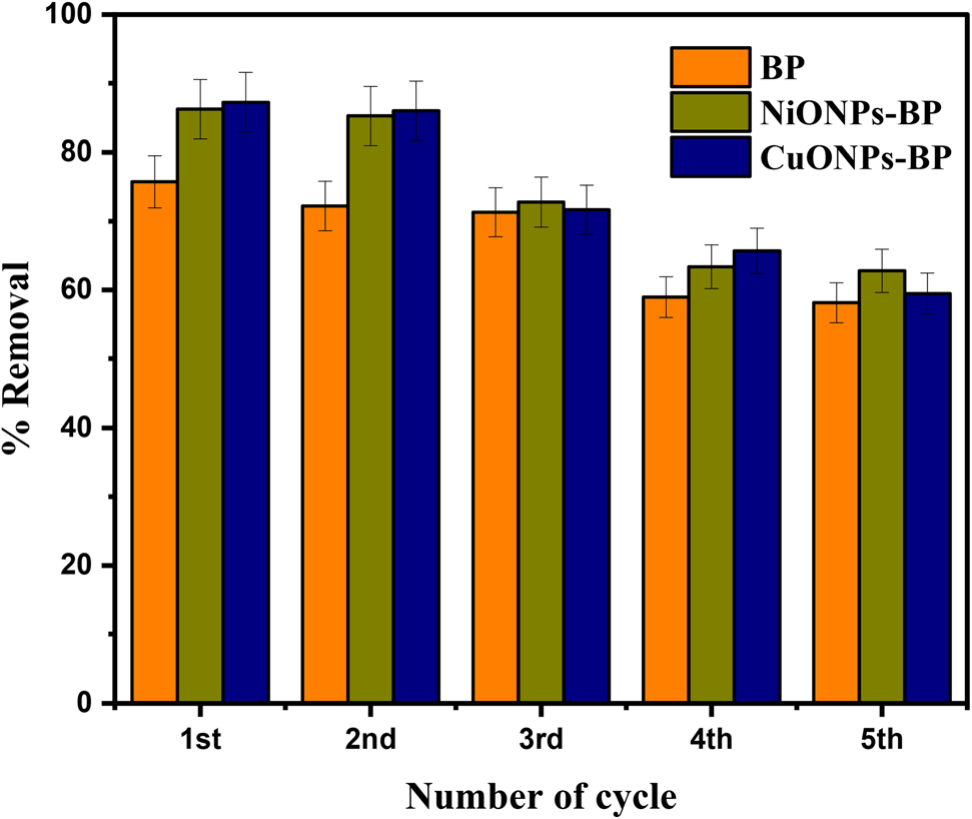
Effect of reuse on the removal of MG on BP, NiONPs-BP and CuONPs-BP.

A reusability study was carried out to evaluate the possibility of recycling NiONPs-BP and CuONPs-BP nanocomposites. The desorption of MG from MG-adsorbed nanocomposites was performed using 0.1 M HNO_3_ as an eluting agent. The dye uptake percentage for BP, NiONPs-BP and CuONPs-BP decreased from 75.72% to 58.14%, 86.22% to 63.79%, and 87.20% to 59.46% after five consecutive cycles, as shown in Fig. 9. After the adsorbent catalyst had been recycled five times, the conversion percentage of MG decreased slightly. This was likely caused by mass loss, incomplete removal of MG pollutant, the decrease of active sites by NiO and CuO escape, and the partial covering of active sites on the catalyst (adsorbent) surface by adsorption.^33^ From an economic and environmentally friendly point of view, it can be said that the studied adsorbents were successful materials after three cycles. The impregnation of NiONPs and CuONPs on the agro waste actually improved the rate of removal of the target pollutant. Sasmal *et al.*^65^ showed that the removal capacity of amylopectin dialdehyde-Schiff base was 74.35% for MG dye, but after the fifth cycle an uptake capacity of 42.30% was reported. Weak binding forces (physical adsorption) were dominant between the dye molecules and adsorbent surface, which promoted high reversibility of the adsorption process.^66^

### 3.8 Proposed mechanism of adsorption

**Fig. 10:**
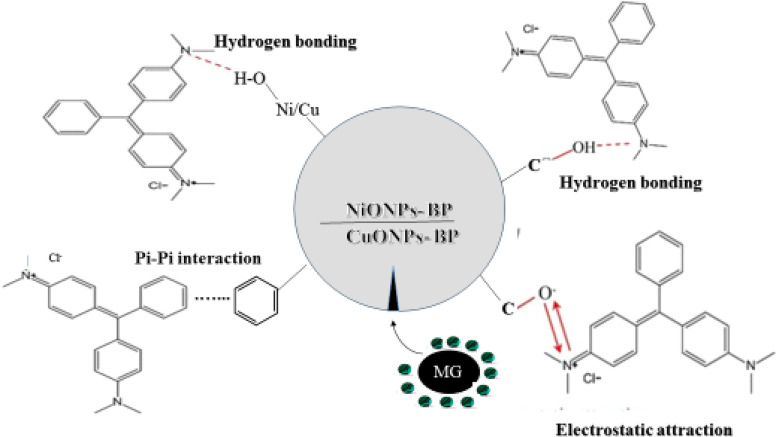
Proposed MG adsorption mechanism onto NiONPs-BP and CuONPs-BP.

In order to describe the adsorption operation for the treatment of dye contaminated industrial effluents, it is crucial to understand the serial mechanism involved in the removal process. A number of variables, including the surface charge index of the interacting species, functional chemical structure, and electrostatic interactions, affect how MG is adsorbed by BP, NiONPs-BP, and CuONPs-BP.^67^ From the displayed SEM images, numerous pores are present for the effective attachment and uptake of MG from aqueous media. In addition, the depicted FTIR spectra (Fig. 1d) of native biomass and the respective nanomaterials before and after MG adsorption showed that O–H, C–O, and C–O were responsible for the uptake of MG dye onto the adsorbents. It has been stated that the presence of the noted functional moieties on the adsorbents showed the good uptake of the target pollutant from the aqueous solution. Due to the abundance of H^+^ for protonation of the sorbents' available functional groups, the surfaces of the nanosorbents remain positively charged at pH < pHpzc. However, at pH > pHpzc, deprotonation facilitates uptake of the cationic dye *via* hydrogen bonding and electrostatic attraction.^68^ Based on the isotherm and thermodynamic studies carried out, it can be proposed that physical adsorption occurred *via* electrostatic attraction.^69^ The active heterogeneous surface sites were actually enhanced by the impregnation of NiO and CuO on the native plants.^70^*n*–π interactions were involved in the aromatic rings of MG as electron acceptors and the O in the carbonyl of the adsorbent surface as an electron contributor.^10^ Hydrogen-bonding interactions, specifically H-bonding, occur between the aromatic rings of MG and the H surface of the hydroxyl groups on the adsorbent. Additionally, dipole–dipole H-bonding interactions exist between the hydrogen surfaces of the hydroxyl groups (acting as donors) on the sorbent and atoms (O and N, acting as acceptors). In addition, being a polyaromatic compound, the benzene rings act as electron-rich zones that can promote *π*–*π* electron donor–acceptor interactions on the adsorbent surface sites before pore diffusion occurs *via* adsorption^71^. The adsorption mechanism is summarized in Fig. 10.

## 4. Conclusion

Bio-based miracle leaves (*Bryophyllum pinnatum*) incorporating nickel oxide and copper oxide were fabricated for the removal of MG from wastewater. The resulting functional groups and intense peaks corresponding to crystalline planes present in the adsorbents revealed an improvement in the surface properties of both unmodified and modified agro waste. The studied raw and nanocoated adsorbents demonstrated significant weight loss upon heating from 100 °C to 400 °C, indicating removal of moisture and decomposition of organic matter. N_2_ adsorption–desorption characteristics indicated significant pore sizes and volumes for the studied nanocomposites with type IV-shaped isotherms, indicating mesoporous structures of the adsorbents. The optimum pH was observed to be in the alkaline range, with the dosage at 0.1 g and contact time at 60 min for all the adsorbents. The maximum removal capacities of BP, NiONPs-BP and CuONPs-BP were 16.26, 28.77 and 13.66 mg g^−1^. The isotherm data for the synthetic dye and metal ion equilibrium bio-sorption showed a good fit for the Freundlich model, with relatively high correlation regression values (*R*^2^ > 0.9) and low statistical error. The pseudo-second order kinetic model accurately matched the experimental data (BP → 0.9946, NiONPs-BP → 0.9997, and CuONPs-BP → 0.9996). Thermodynamic studies suggested the physical process of the adsorption mechanism and negative values of Δ*G*°, Δ*H*° and Δ*S*° for MG dye removal showed that the adsorption operation is spontaneous, and exothermic with reduced degree of randomness for BP and CuONPs-BP. The nanocomposites (NiONPs-BP and CuONPs-BP) exhibited reliable reusability after five repeated adsorption cycles compared to BP. The hydroxyl, carboxyl, amine and ether groups dominated the adsorbent binding sites for the MG dye with an underlying mechanism that involved an electrostatic relationship, ion exchange, complexation, *π*–*π* interactions, hydrogen bonding, pore diffusion and micro precipitation. The complex formation of nickel oxide and copper oxide with the agro waste aid in improving the adsorption efficiency with the potential capability to remove MG dye from polluted water.

## Author contributions

Umeh, C. T.: conceptualization, methodology, software; Nduka, J. K., Akpomie K. G.: supervision, writing-reviewing and editing; Obi, A. I., Akanbi, M. N.: software and reviewing.

## Conflicts of interest

The authors declare no financial competing interest.

## Supplementary Material

RA-015-D5RA05373K-s001

## Data Availability

Data have been included in the article and SI, while some will be made available on request. Supplementary information: Equation of the isotherm models and kinetics as well. See DOI: https://doi.org/10.1039/d5ra05373k.
