# Supplementary material for: Remediation of malachite green-laden wastewater by plant-loaded metal oxide nanocomposites
Source: RSC Adv. 2025 Sep 11;15(39):32942–55. doi: 10.1039/d5ra05373k (PMC12423763; doi:10.1039/d5ra05373k)
Supplement: RA-015-D5RA05373K-s001 [file RA-015-D5RA05373K-s001.pdf]

This journal is © The Royal Society of Chemistry 2025

Supplementary file

Isotherm model

The adsorption isotherm was modeled with linear form of Langmuir, Freundlich, Temkin, Dubinin and Radushkevich (D–R) and Sips isotherms as represented in equations 1 – 5 to fit the equilibrium data for MG adsorption at room temperature.

$$\frac{C_e}{q_e} = \frac{1}{q_{mK_L}} + \frac{C_e}{q_m} \quad 1$$

$$\text{Log } q_e = \log K_F + \frac{1}{n} \log C_e \quad 2$$

$$qe = B\, lnK_T + B\, lnC_e \quad 3$$

$$\ln qe = \ln q_m - K_{DR}\varepsilon^2 \quad 4$$

$$\varepsilon = RT\ln\,[1 + \frac{1}{C_e}]$$

$$E = \frac{1}{\sqrt{2K_{DR}}}$$

$$ln\left(\frac{q_e}{q_{max} - q_e}\right) = \frac{1}{n} ln\, C_e + \ln K_S \quad 5$$

Where qe is the amount of adsorbate adsorbed per unit mass of adsorbent (mg/g), q_m is the maximum adsorption capacity (mg/g), Ce is the equilibrium concentration of metal ion and dye in the solution (mg/L), K_L is Langmuir constant related to the affinity of binding sites (L/mg), and is the measure of adsorption energy. The parameters K_F and n are Freundlich constants which can be regarded as the capacity and intensity of adsorption, respectively. B is the Temkin constant related to the heat of adsorption (kJ/mol), and K_T (L/mg) was the equilibrium binding constant relating to the maximum binding energy. K_{DR} (mol^2/kJ^2) is a constant related to the mean adsorption energy; and ε is the Polanyi potential, *R* (8.314 J/mol**.** K) is universal gas constant, *T* (Kelvin) is absolute temperature, *E* (kJ/mol) is mean free energy of adsorption. Ks is Sips equilibrium constant (L/mg), and 1/n is Sips model exponent. If the value for 1/n is < one, it shows that it is a heterogeneous adsorbent, while values closer to or even one indicates that the adsorbent has relatively more homogeneous binding sites.

Looking at Langmuir model, to evaluate the favorability of the adsorption system, the isotherm was categorized with separation factor indicated in equation 7.

$$R_L = \frac{1}{1 + K_L C_o}$$ 6

where C_o (mg/L) is the highest initial concentration of dye and K_L(L/mg) is the Langmuir constant. The parameter R_L indicates the shape of the isotherm to be either unfavorable ($R_L > 1$), linear ($R_L = 1$), favorable ($0 < R_L < 1$) or irreversible ($R_L = 0$).

Kinetic model

The kinetics of MG adsorption on BP, NiONPs-BP and CuONPs-BP were fitted to five models including pseudo-first order (PFO), pseudo-second order (PSO), Elovich, intraparticle diffusion (IPD) and Liquid film diffusion (LFD) models to identify and understand the adsorption mechanism of the selected adsorbate uptake onto adsorbent surface. The linear fittings of the aforementioned kinetic models are represented using the following equations:

$$\log(qe - qt) = loq\ qe - \frac{K_1 t}{2.303}$$ 7

$$h_1 = k_1 q_e$$

$$\frac{t}{qt} = \frac{1}{k_2 qe^2} + \frac{t}{qe}$$ 8

$$h_2 = k_2 qe^2$$

$$qt = \frac{1}{\beta}\ln(\alpha\beta) + \frac{1}{\beta}\ln(t)$$ 9

$$qt = K_{id} t^{1/2} + C$$ 10

$$\ln(1 - F) = -K_{LFD} t + C$$ 11

$$F = \frac{[q_t]}{[q_e]}$$

where qe and qt are the amount of compound adsorbed at equilibrium and at any time t (mg/g); k_1 (min^{-1}), k_2 (g/mg min), K_{id} (mg/g/min$^{1/2}$) and K_{LFD} (min^{-1}) are the equilibrium rate constants of pseudo-first-order, second-order, intraparticle diffusion and liquid film diffusion adsorption; C corresponds to the intercept of IPD and LFD equation; The constant F represents fractional

attainment to equilibrium, α is initial sorption rate (mg/g min) and β is a desorption constant related to the extent of surface coverage and activation energy for chemisorption (g/mg).

Thermodynamics

The following equations were used for thermodynamic study.

$$\Delta G^o = -RTlnK_C = \Delta H^o - T\Delta S^o \quad 12$$

$$lnK_C = -\left(\frac{\Delta H^o}{RT}\right) + \left(\frac{\Delta S^o}{R}\right) \quad 13$$

$$lnK_C = \frac{q_e}{c_e} \quad 14$$

where $\Delta G°$ is change in standard Gibbs free energy, R is a universal gas constant 8.314 J/ (mol. K), and T = Temperature, ΔH°, ΔG°, and ΔS° denotes change in enthalpy, free energy, entropy, respectively, and K_C is adsorption thermodynamic equilibrium constant.

Error analysis

To estimate a better fit or accuracy in determining which of the isotherm models is best to describe the adsorption process, three statistical error function were utilized as shown in the following equations**.**

$$\text{Sum square error (SSE)} = \sum_{i=1}^{n} (q_{e,cal} - q_{e,exp})_i^2 \quad 15$$

$$\text{Sum of absolute error (EABS)} = \sum_{i=1}^{n} |q_{e,exp} - q_{e,cal}| \quad 16$$

$$\text{Chi-square test } (\chi^2) = \sum_{i=1}^{n} \frac{(q_{e,exp} - q_{e,cal})^2}{q_{e,exp}} \quad 17$$

Where $q_{e,exp}$ is the experimental value while $q_{e,cal}$ is the calculated value from the isotherm models and n is the number of observations in the experiment
